# Analytical and quasi-Bayesian methods as development of the iterative approach for mixed radiation biodosimetry

**DOI:** 10.1007/s00411-018-0745-6

**Published:** 2018-06-04

**Authors:** Iwona Słonecka, Krzysztof Łukasik, Krzysztof W. Fornalski

**Affiliations:** 1 Central Laboratory for Radiological Protection, Konwaliowa 7, 03-194 Warszawa, Poland; 2 Faculty of Physics, Warsaw University of Technology, Warszawa, Poland; 3 Ex-Polon Laboratory, Łazy, Poland; 4 PGE EJ 1 Sp. z o. o., Warszawa, Poland

**Keywords:** Biological dosimetry, Bayesian, Nuclear accident, Cytogenetics, Radiation, Biodosimetry, Dose assessment

## Abstract

The present paper proposes two methods of calculating components of the dose absorbed by the human body after exposure to a mixed neutron and gamma radiation field. The article presents a novel approach to replace the common iterative method in its analytical form, thus reducing the calculation time. It also shows a possibility of estimating the neutron and gamma doses when their ratio in a mixed beam is not precisely known.

## Introduction

Radiation biodosimetry refers to the analysis of biological changes in a particular tissue after exposure to ionizing radiation, and allows for an estimation of absorbed doses in an exposed person based on this analysis. The quantifying measurement of chromosome alterations, mainly dicentrics in lymphocytes, is the most widely used method for biological dose assessment (IAEA 2011). The aim of radiation biodosimetry by cytogenetic methods is to calculate doses and the associated confidence intervals to exposed (or suspected to have been exposed) individuals after a radiation accident or incident. Assessing the absorbed dose based on the dicentric assay in peripheral blood lymphocytes is a very sensitive method that can be used when measured doses are not available. The analysis is performed in the lymphocytes during their first mitosis after radiation exposure. The advantage of the method is the low spontaneous frequency of dicentric chromosome aberrations in healthy individuals, indicating that this indicator is caused mainly by ionizing radiation.

The process of dose assessment through a dicentric assay requires the presence of dose–response calibration curves. Such curves are produced by exposing human blood in vitro to several doses of radiation under carefully controlled conditions. It is implicitly assumed that in vivo and in vitro irradiations of peripheral blood lymphocytes produce similar alterations in the cells. Accordingly, the dicentric frequency observed in vivo can be converted to absorbed dose by comparing it with the dose–response calibration curve obtained in vitro. The shape of a dose–response curve is influenced by the linear energy transfer, LET, for any particular radiation quality. For low-LET radiation, the relation between absorbed dose and dicentric chromosome frequency can be expressed as a combination of linear and quadratic terms (Eq. 1), while for high-LET radiation the dose dependence is linear (Eq. 2). The linear and quadratic terms are consistent with the single- and two-track model of dicentric formation by both radiation qualities (IAEA 2011; Kellerer and Rossi 1978):

1 $${Y_g}\left( {{D_g}} \right)={Y_0}+\beta ~{D_g}~+\gamma ~{D_g}^{2},$$

2 $${Y_n}\left( {{D_n}} \right)={Y_0}+\alpha ~{D_n},$$

where *Y*_*x*_ (*x* = *g* is for gamma and *x* = *n* for neutron radiation) is the frequency of dicentrics, *D*_*x*_ is the absorbed dose, *Y*_*0*_ is the background frequency of dicentrics (dose independent), *α* and *β* are the linear coefficients, and *γ* is the quadratic coefficient.

In the case of exposure to mixed neutron and gamma radiation, *n* + *γ* (e.g., after a nuclear reactor accident), biological dosimetry is more complex than in the case of exposure to a single type of radiation. Mixed radiation fields are composed of particles with varying biological effectiveness, which have different biological effects on the body. Therefore, there is a strong need to calculate not only the total absorbed dose, but also its components, *D*_*n*_ and *D*_*g*_, separately. Unfortunately, there is no visible difference between dicentrics induced by the two types of radiation, and therefore, it is not possible to directly discriminate between dicentrics produced by gamma radiation and those produced by neutrons. Discrimination is only possible by quantifying the separate dose components and using the additivity assumption in the production of chromosomal damages (IAEA 2011; Kellerer and Rossi 1978).

Dose estimation using biodosimetry methods for a mixed neutron and gamma radiation field can be found in the literature (IAEA 2011; Brame and Groer 2003; Szłuińska et al. 2005; Fornalski 2014). The most common approaches are the classical iterative method, promoted widely by the International Atomic Energy Agency (IAEA 2011), and the increasingly used Bayesian methods (Brame and Groer 2003; Ainsbury et al. 2013a, b; Pacyniak et al. 2014). The iterative method is adopted in many accredited laboratories worldwide, including the Central Laboratory for Radiological Protection (CLOR), Poland. This method is generally well-known due to its simplicity and relative accuracy. Bayesian methods are also becoming more and more popular. However, they are not yet commonly used probably due to the much more advanced mathematical approach required. Thus, some intermediate methods, which will improve the iterative approach without being as complex as the Bayesian approach, are developed and tested in the present paper.

## Materials and methods

### Assessment of neutron and gamma doses

In practice, it is assumed that the number of dicentrics in an analyzed sample is characterized by a Poisson distribution, and that the observed alterations are the sum of those induced by neutron and gamma radiation. Thus, the dose–response relationship for a mixed neutron and gamma radiation field may be described as a combination of Eqs. 1 and 2:

3 $${Y_{n+g}}\left( {{D_n},{D_g}} \right)={Y_0}+\alpha ~{D_n}~+~\beta ~{D_g}~+\gamma ~{D_g}^{2}~ \equiv ~{y_f}=\frac{u}{w},$$

which is usually called a combined linear-quadratic equation for the frequency of chromosome aberrations *y*_*f*_ after irradiation by a mixed neutron and gamma radiation field with doses *D*_*n*_ and *D*_*g*_. Parameters *Y*_*0*_, *α, β* and *γ* are usually found as a result of a regression analysis (defining the so-called calibration curve). The parameter *y*_*f*_ can be written as a ratio of *u*/*w*, where *u* represents the number of chromosome aberrations and *w* is the number of cells in the analyzed sample.

Having the fit parameters of the calibration curves, (*Y*_*0*_, *α, β*, and *γ*), the *Y*(*D*_*x*_) functions can be used to estimate the doses (*D*_*x*_) and/or the frequency of chromosome aberrations (*Y*_*x*_) after exposure to gamma radiation, neutron radiation, or to a mixed neutron and gamma radiation field. In cases when the ratio of neutron to gamma absorbed dose is estimated by physical methods (Eq. 4):

4 $$\rho =\frac{{{D_n}}}{{{D_g}}},$$

it is possible to calculate the separate neutron and gamma radiation doses using the iterative method (IAEA 2011) mentioned earlier. In this method, the doses and the chromosome aberration frequencies are estimated by a manual iterative approach, meaning that the values are precisely determined.

### Iterative method

The iterative method involves performing several series of calculations using the same input data. Each consecutive series gives more accurate results, until the results of the next steps do not change significantly any more. In this method, it is initially assumed that all aberrations found in the analyzed sample, *y*_*f*_, are caused by neutron radiation, i.e., using the recurrence relation, ${Y_{{n_{i=0}}}}={y_f}$. Thus, the neutron dose can be defined directly from Eq. 2, as:

5 $${D_{{n_{i+1}}}}=\frac{{{Y_{{n_i}}} - {Y_0}}}{\alpha }.$$

The dose from gamma radiation in the (*i* + 1)^th^ step, ${D_{{g_{i+1}}}}$, is calculated with the use of the actual ${D_{{n_{i+1}}}}$ value and the neutron/gamma dose ratio, *ρ*, according to Eq. 6:

6 $${D_{{g_{i+1}}}}=\frac{{{D_{{n_{i+1}}}}}}{\rho },$$

also formulated as the recurrence equation. With information about the gamma dose, the dicentric frequency due to gamma radiation, ${Y_{{g_{i+1}}}}$, can be obtained from Eq. 1:

7 $${Y_{{g_{i+1}}}}={Y_0}+\beta {D_{{g_{i+1}}}}+\gamma D_{{{g_{i+1}}}}^{2}.$$

The dicentric frequency caused by neutrons (${Y_{{n_{i+1}}}}$) is then obtained (Eq. 8) by subtracting the gamma dicentric frequency from the measured aberrations, *y*_*f*_.

8 $${Y_{{n_{i+1}}}}={y_f} - {Y_{{g_{i+1}}}}.$$

The neutron dose is estimated using Eq. 5 and the above steps are repeated using recurrence equations from Eqs. 5–8. With this algorithm, all parameters are calculated iteratively until their values are stable (${D_{{x_i}}} \cong {D_{{x_{i - 1}}}}$).

Obviously, the more repetitions done, the more accurate the achieved values will be. All the fitting parameters of the calibration curves (*Y*_*0*_, *β, γ*, and *α*) as well as the ratio of doses (*ρ*) should be calculated in advance. However, a significant disadvantage of the iterative method is the large effect of the propagation of uncertainty. The presented iterative algorithm is usually time-consuming and requires many repetitions to obtain final results. Hence, it was proposed to transform the iterative method into its analytical description, which was originally introduced by Fornalski (2014).

### Analytical method

The analytical method (Fornalski 2014; Pacyniak et al. 2015) was proposed to automate the iterative algorithm used for calculating the absorbed doses in mixed radiation fields and exclude the propagation of uncertainty effect. In this approach, the neutron/gamma dose ratio, *ρ* (Eq. 4), must be known. However, because *ρ* varies from zero to infinity, it can conveniently be replaced by *θ* (Eq. 9), which corresponds to the contribution of the gamma dose to the total dose and is more practical in use than *ρ*. It is normalized to the range of [0, 1] and is defined as (Brame and Groer 2003):

9 $$\theta =\frac{{{D_g}}}{{{D_g}+{D_n}}}=\frac{1}{{\rho +1}}.$$

With Eqs. 3 and 9, *D*_*g*_ and *D*_*n*_ can be simply calculated as a function of *θ* (Fornalski 2014):

10 $$\left\{ {\begin{array}{*{20}{c}} {{D_g}\left( \theta \right)=~\frac{{\sqrt {{{\left( {\alpha \frac{{1 - \theta }}{\theta }+\beta } \right)}^2}+4\gamma \left( {{y_f} - ~{Y_0}} \right)} - \left( {\alpha \frac{{1 - \theta }}{\theta }+\beta } \right)}}{{2\gamma }}} \\ {{D_n}\left( \theta \right)=\frac{{1 - \theta }}{\theta }{D_g}\left( \theta \right)} \end{array}} \right..$$

**Fig. 1:**
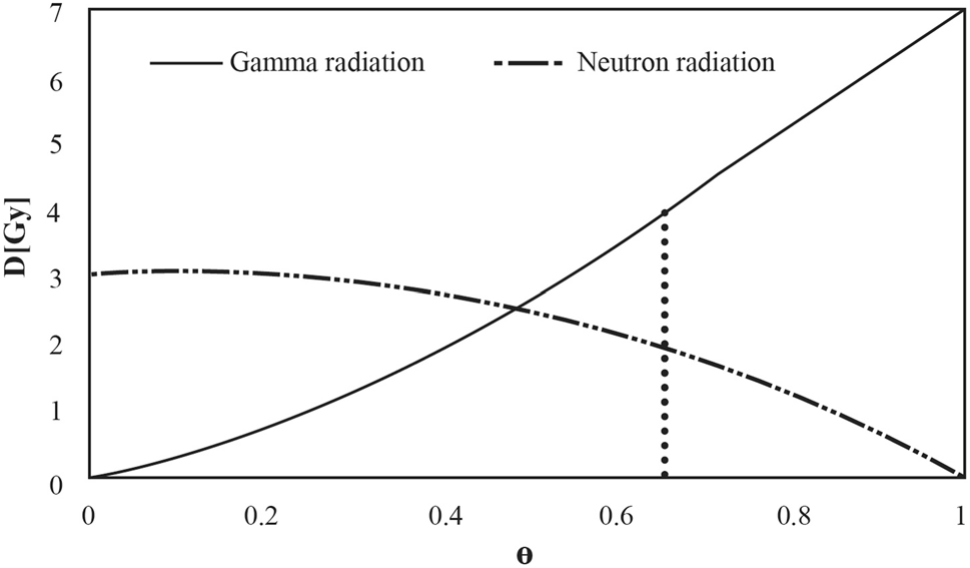
Distributions of gamma and neutron doses for a sample with 53 dicentrics in 19 analyzed cells obtained with the analytical method. *θ* = 0.653 is marked with a dotted line

It is assumed that all constants (*α, β, γ, y*_*f*_, *Y*_*0*_ and *θ*) are precisely known from experimental data with proper uncertainties. The solution of Eq. 10 can be presented graphically in terms of dose and *θ* (Fig. 1).

Finally, the uncertainties associated with the estimated doses, ${\sigma _{{D_x}}}$, in the presented method can be calculated using the delta method (as it was used in the current work):

11 $${\sigma _{{D_x}}}=\sqrt {{{\left( {\frac{{\partial {D_x}}}{{\partial \alpha }}} \right)}^2}\delta _{\alpha }^{2}+{{\left( {\frac{{\partial {D_x}}}{{\partial \beta }}} \right)}^2}\delta _{\beta }^{2}+{{\left( {\frac{{\partial {D_x}}}{{\partial \gamma }}} \right)}^2}\delta _{\gamma }^{2}+{{\left( {\frac{{\partial {D_x}}}{{\partial {y_f}}}} \right)}^2}\delta _{{{y_f}}}^{2}+{{\left( {\frac{{\partial {D_x}}}{{\partial {Y_0}}}} \right)}^2}\delta _{{{Y_0}}}^{2}+{{\left( {\frac{{\partial {D_x}}}{{\partial \theta }}} \right)}^2}\delta _{\theta }^{2}} ,$$

where *x* = {*g, n*}, and ${\delta _\varepsilon }=\left\{ {{\sigma _\alpha },~{\sigma _\beta },~{\sigma _\gamma },~{\sigma _{{y_f}}},~{\sigma _{{Y_0}}},~{\sigma _\theta }} \right\}$ are uncertainties of parameters *ε* = {*α, β, γ, y*_*f*_, *Y*_*0*_, *θ*}. Uncertainties of *D*_*g*_ and *D*_*n*_ can be calculated from Eq. 11 finding partial derivatives of *D*_*x*_.

### Quasi-Bayesian (Q-B) method

When the ratio of doses is not precisely known, the iterative and analytical methods cannot be used. In this case, the probability density function (PDF) can be utilized to estimate the most probable value of *θ* (or *ρ*). Such a PDF, which is widely used in Bayesian statistics, is called a prior probability *p(θ)* or *p(ρ)*. In the most common cases it can be approximated by a Gaussian distribution for *θ* (or *ρ*) with a standard deviation of *σ*_*θ*_ (or *σ*_*ρ*_), for example:

12 $$p(\theta )=\frac{1}{{\sqrt {2\pi } {\sigma _\theta }}}\exp \left[ { - \frac{{{{\left( {\theta - \hat {\theta }} \right)}^2}}}{{2\sigma _{\theta }^{2}}}} \right]~,$$

where $\hat {\theta }$ represents the expected value of *θ* with the uncertainty *σ*_*θ*_. The Gaussian distribution is usually selected because the estimation of *θ* (or *ρ*) is done using the classical Gaussian regression method. However, one can also use other priors reflecting the knowledge on *θ* (or *ρ*); this issue will be discussed later.

Having sufficient information on the dose ratio, one can try to estimate the neutron and gamma doses, as well as the dicentric frequencies. To enhance the classical method for the possibility that *θ* is not given by the value, but by the prior probability density function (PDF), which is the first step to Bayesian reasoning, one has to transform classical relationships into probability distributions (put this way the method can also be called the Bayesian–frequentist hybrid method). However, contrary to the full-Bayesian method, the multiplication of prior and likelihood functions is avoided. Thus, the proposed quasi-Bayesian method is somewhere between the analytical (classical) method and the full-Bayesian method.

The proposed quasi-Bayesian method uses fixed values for the dose response parameters *α, β, γ*, and *Y*_*0*_, which can be assessed beforehand by means of maximum likelihood estimation, the least squares method, or even by the robust Bayesian regression method (Fornalski 2014). The Q-B method does not use the uncertainties of those parameters directly in the dose calculations, but only in the assessment of dose uncertainty. This approach is much easier than using probability densities of all parameters [which are used in the full-Bayesian method (Brame and Groer 2003)], and can be efficiently utilized in the case when the parameter values are known from earlier estimations.

In practice, Eqs. 3 and 9 must be solved to find distributions of *θ* in a mixed neutron and gamma radiation field (Eq. 13) (Fornalski 2014):

13 $$\left\{ {\begin{array}{*{20}{c}} {{\theta _g}\left( {{D_g}} \right)=\frac{{{D_g}}}{{{D_g}+\frac{1}{\alpha }\left( {{y_f} - ~{Y_0} - \beta {D_g} - \gamma {D_g}^{2}} \right)}}} \\ {{\theta _n}\left( {{D_n}} \right)=\frac{{\sqrt {{\beta ^2} - 4\gamma \left( {~{Y_0}+\alpha {D_n} - {y_f}} \right)} - \beta }}{{\sqrt {{\beta ^2} - 4\gamma \left( {~{Y_0}+\alpha {D_n} - {y_f}} \right)} - \beta +2\gamma {D_n}}}} \end{array}} \right..$$

The two different designations of *θ* result from the fact that *θ* is not precisely described by its value, but by the prior probability function. Thus, using changing variables, the probability distribution of the dose can be generally written as:

14 $$P\left( {{D_x}} \right)~=p\left( {{\theta _x}({D_x})} \right) \cdot \frac{{\partial {\theta _x}}}{{\partial {D_x}}} \cong p\left( \theta \right) \cdot {\text{const.}}$$

Changing variables (Eq. 14) jointly with the expressions in Eq. 12 (which exemplifies a potential PDF for *θ;* here it is a normal distribution, but other distributions can be used as well, as seen below) and Eq. 13 gives a system of two probability distributions for *D*_*g*_ and *D*_*n*_ (Eq. 15).

15 $$\left\{ {\begin{array}{*{20}{c}} {P({D_g})=\frac{1}{{\sqrt {2~\pi } {\sigma _\theta }}}\exp \left[ { - \frac{{{{\left( {\frac{{{D_g}}}{{{D_g}+\frac{1}{\alpha }\left( {{y_f} - {Y_0} - \beta {D_g} - \gamma D_{g}^{2}} \right)}}~ - ~\hat {\theta }} \right)}^2}}}{{2~\sigma _{\theta }^{2}}}} \right]} \\ {P({D_n})=\frac{1}{{\sqrt {2~\pi } {\sigma _\theta }}}\exp \left[ { - \frac{{{{\left( {\frac{{\sqrt {{\beta ^2} - 4\gamma \left( {{Y_0}+\alpha {D_n} - {y_f}} \right)} - \beta }}{{\sqrt {{\beta ^2} - 4\gamma \left( {{Y_0}+\alpha {D_n} - {y_f}} \right)} - \beta +2\gamma {D_n}}}~ - ~\hat {\theta }} \right)}^2}}}{{2~\sigma _{\theta }^{2}}}} \right]} \end{array}} \right..$$

Finally, dose *D*_*x*_ can be estimated from the maximum of the distributions given in Eq. 15or calculated from the first derivative equation:

16 $$\frac{{{\text{d}}P({D_x})}}{{{\text{d}}{D_x}}}=0.$$

The dose uncertainties ${\sigma _{{D_x}}}$, obtained in the presented method can be assessed using the approach described for the analytical method (Eq. 11). In this case, one needs to determine the equation for *D*_*x*_ directly from Eq. 15 (or generally from Eq. 14) using numerical methods, because an analytical solution is usually impossible. After that, the calculated *D*_*x*_ is included into Eq. 11 to get the uncertainty value, finding partial derivatives of *D*_*x*_. This approach was used in the current work to calculate all uncertainties in the quasi-Bayesian method (see Table 1).

**Table 1:** Comparison of data from the literature (biological, based on the classical iterative approach, and reference values determined experimentally by the use of sophisticated physical instruments) with results obtained with the analytical and quasi-Bayesian methods described in the present work

Source of data	*Y*_n_* [dic/cell]	*Y*_g_* [dic/cell]	*ρ*	Cells	Dicentrics	Biological doses		Reference doses		Analytical		Quasi-Bayesian: Gaussian prior	
						*D*_*n*_ [Gy]	*D*_*g*_ [Gy]	*D*_*n*_ [Gy]	*D*_*g*_ [Gy]	*D*_*n*_ [Gy]	*D*_*g*_ [Gy]	*D*_*n*_ [Gy]	*D*_*g*_ [Gy]
IAEA	*Y* = 0.0005 + 0.832D	*Y* = 0.0005 + 0.0164D + 0.0492D^2^	0.67	100	120	1.21	1.82	Unknown		1.21 ± 0.15	1.81 ± 0.25	1.21 ± 0.15	1.81 ± 0.25
HPA	*Y* = 0.0005 + 0.83D	*Y* = 0.0005 + 0.014D + 0.076D^2^	0.67	100	100	1.00	1.50	Unknown		0.98 ± 0.13	1.47 ± 0.20	0.98 ± 0.13	1.47 ± 0.20
NRPB	*Y* = 0.835D	*Y* = 0.0142D + 0.0759D^2^	5.78	34	100	3.50 ± 0.30	0.60 ± 0.06	3.42	0.59	3.48 ± 0.70	0.60 ± 0.15	3.48 ± 0.70	0.60 ± 0.15
			1.87	40	108	3.00 ± 0.30	1.59 ± 0.15	3.42	1.83	2.98 ± 0.52	1.59 ± 0.30	2.98 ± 0.53	1.59 ± 0.31
			0.64	28	85	2.30 ± 0.30	3.70 ± 0.40	2.60	4.04	2.35 ± 0.40	3.67 ± 0.62	2.35 ± 0.40	3.67 ± 0.62
			0.53	10	37	2.40 ± 0.40	4.60 ± 0.80	2.60	4.89	2.44 ± 0.62	4.59 ± 1.16	2.43 ± 0.62	4.59 ± 1.16
IRSN	*Y* = 0.9D	*Y* = 0.023D + 0.054D^2^	5.78	50	125	2.70 ± 0.20	0.46 ± 0.04	3.42	0.59	2.75 ± 0.48	0.48 ± 0.11	2.75 ± 0.48	0.48 ± 0.11
			1.87	55	202	3.80 ± 0.30	2.04 ± 0.14	3.42	1.83	3.78 ± 0.56	2.02 ± 0.34	3.78 ± 0.56	2.02 ± 0.34
			0.64	14	36	2.10 ± 0.40	3.30 ± 0.60	2.60	4.04	2.12 ± 0.55	3.31 ± 0.87	2.12 ± 0.55	3.31 ± 0.87
			0.53	19	53	2.10 ± 0.30	3.90 ± 0.50	2.60	4.89	2.08 ± 0.44	3.92 ± 0.83	2.08 ± 0.44	3.92 ± 0.83
A	*Y* = 0.001 + (0.603 ± 0.079)D	*Y* = 0.001 + (0.0187 ± 0.0056)D + (0.0527 ± 0.0046)D^2^	0.60	61	129	2.20	3.70	1.83	3.03	2.21 ± 0.31	3.68 ± 0.51	2.21 ± 0.31	3.68 ± 0.51
			2.08	278	185	1.10	0.50	0.85	0.41	1.06 ± 0.17	0.51 ± 0.09	1.06 ± 0.17	0.51 ± 0.09
			2.08	50	81	2.50	1.20	1.80	0.87	2.52 ± 0.53	1.22 ± 0.27	2.52 ± 0.53	1.21 ± 0.27
B	*Y* = 0.001 + (0.638 ± 0.018)D	*Y* = 0.001 + (0.0371 ± 0.0085)D + (0.0547 ± 0.0039)D^2^	0.60	46	114	2.40	3.90	1.83	3.03	2.35 ± 0.33	3.91 ± 0.54	2.35 ± 0.33	3.91 ± 0.54
			2.08	151	112	1.10	0.50	0.85	0.41	1.11 ± 0.14	0.53 ± 0.08	1.11 ± 0.14	0.53 ± 0.08
			2.08	67	128	2.50	1.20	1.80	0.87	2.76 ± 0.40	1.33 ± 0.22	2.76 ± 0.40	1.33 ± 0.22
C	*Y* = 0.001 + (0.832 ± 0.031)D	*Y* = 0.001 + (0.0128 ± 0.0031)D + (0.0640 ± 0.0022)D^2^	0.60	100	186	1.60	2.70	1.83	3.03	1.63 ± 0.18	2.71 ± 0.31	1.63 ± 0.18	2.71 ± 0.31
			2.08	100	81	1.00	0.50	0.85	0.41	0.95 ± 0.14	0.46 ± 0.08	0.95 ± 0.15	0.46 ± 0.08
			2.08	100	144	1.70	0.80	1.80	0.87	1.67 ± 0.22	0.80 ± 0.13	1.67 ± 0.22	0.80 ± 0.13

Uncertainties represent 95% confidence intervals. * Presented uncertainties were taken from the original literature. If they were not available, it was assumed that the following standard values could be used to calculate the dose uncertainties in the analytical and QB methods: *σα* = 0.03, *σβ* = 0.003, *σY*_0_ = 0.0001, *σγ* = 0.0016. Data were taken from IAEA (2011) (in table marked as IAEA—International Atomic Energy Agency), Szłuińska et al. (2005) (HPA—Health Protection Agency), Voisin et al. (1997) (NRPB—National Radiation Protection Broad and IRSN—Institut de Radioprotection et de Sûreté Nucléaire, formerly IPSN—Institut de Protection et de Sûreté Nucléaire), and Voisin et al. (2004) (*A, B, C*—unknown laboratories)

#### Prior distribution functions

The PDF used in the Q-B method (see example given in Eq. 12) should reflect the actual knowledge about the dose ratios. To select the proper PDF, information about *θ* (or *ρ*), such as, for example, the expected value, needs to be considered. Based on all the available information, the prior function (in some cases with its scale and shape parameters) should be used to maximize the PDF for the considered *θ* (*ρ*) parameter. As it was mentioned earlier, the most often used Gaussian distribution (Eq. 12) may be substituted by other PDFs. For the present work, different functions were tested, both symmetrical and unsymmetrical ones. In special cases, even a non-informative prior can be used, which does not specify any exact information, but instead only defines a very general way of parameter search. This approach can be used if detailed information about the dose ratio is missing. Non-informative priors can also be used in situations when it is assumed that one type of radiation contributes most significantly to the investigated biological effect, but the knowledge about the percentage value of *ρ* does not exist **(**Pacyniak et al. 2015**)**.

All results presented below were obtained with the Gaussian PDF (Eq. 12).

### Statistical test *E*_*n*_

To verify the accuracy of the proposed methods, results were tested using *E*_*n*_ test (Pacyniak et al. 2015).

17 $${E_n}=\frac{{\left| {{D_{{\text{ref}}}} - {D_M}} \right|}}{{\sqrt {\sigma _{{{\text{ref}}}}^{2}+\sigma _{M}^{2}} }},$$

where *D*_ref_ is the dose from the reference source (here biological doses), *D*_*M*_ is the dose assessed by the proposed method, *σ*_ref_ and *σ*_*M*_ are their uncertainties, respectively (see Table 1). In the case when *σ*_ref_ is not available, it was assumed here that *σ*_*ref*_ = *10% D*_ref_, which is typical in this type of biodosimetric assessments. If *E*_*n*_ ≤ 1, the result is satisfactory. Any result is classified as an outlier if the *E*_*n*_ value is greater than 1.

### Computational program

As part of the project, a mobile phone application including the described algorithm was created. The application is designed for devices with the Android operating system and was written using the Android Studio Integrated Development Environment (IDE). The graphs were implemented using the GraphView open-source library.

The structure of the program is relatively simple, as it relies on the implementation of a basic Android graphics component (TabLayout), which allows the user to choose between one of the methods discussed above (iterative, analytical or quasi-Bayesian). The program allows the user to change the dose–response curve parameters and select one of the methods to assess absorbed dose. This can be done by selecting the dose–response curves tab from the drop-down menu located at the top right-hand corner of the menu. An important feature of the app is the possibility of drawing the prior function (for the Bayesian and quasi-Bayesian methods), allowing the user to quickly and efficiently choose the distribution and its parameters.

**Fig. 2:**
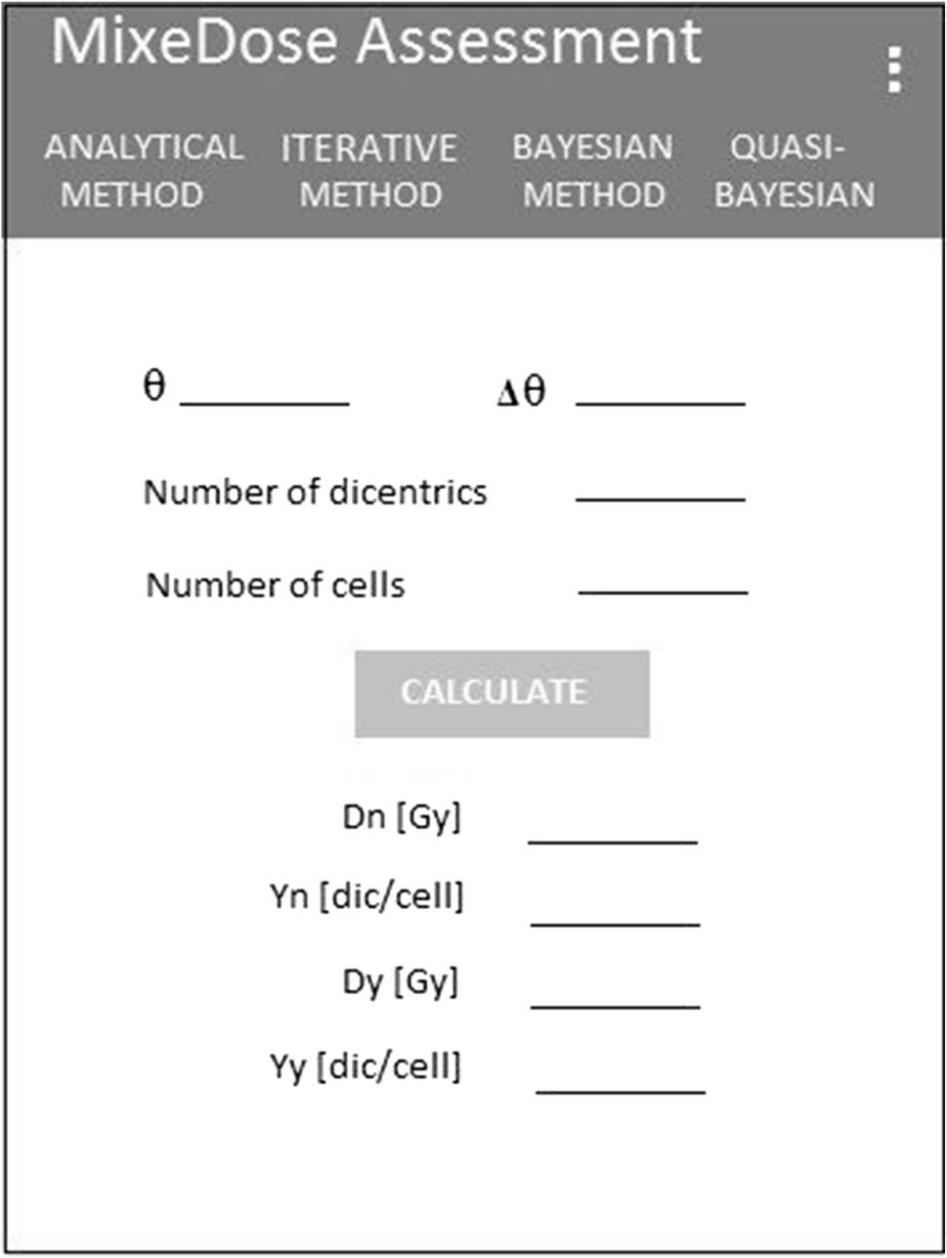
Screenshot of the computational program which uses the presented methods

The program includes a user-friendly interface, which allows the automation of all calculations. The calculation algorithms for determining absorbed doses were implemented in accordance with the theory. There were no approximations done in any of the methods, only the final results displayed on the screen are rounded (with a 1/1000 precision). The program is constantly being improved, and work is underway to enhance its responsiveness. It is available for testing on the website: http://www.clor.waw.pl/publikacje.html. A sample screenshot of the program is shown in the Fig. 2.

## Results

In this work, a number of published dose estimates from biodosimetry studies have been used for comparison with the dose estimates obtained by means of the proposed analytical and quasi-Bayesian methods. More specifically, to verify the validity and credibility of these methods, mixed gamma and neutron radiation doses were calculated using data from the peer-reviewed literature: from IAEA (2011), Szłuińska et al. (2005), Voisin et al. (1997), and Voisin et al. (2004). The fitted coefficients of the dose–response curves, the values for *θ* or *ρ*, the number of analyzed cells and the number of dicentrics measured in the sample were used to make the calculations. Table 1 shows the corresponding data and biological doses for gamma and neutron radiation from the literature mentioned above, as well as the results of the dose assessments obtained by applying the described analytical and quasi-Bayesian methods. Note that the biological doses taken from the literature were obtained using the iterative procedure. Reference values of doses and the neutron/gamma dose ratios were determined experimentally by the use of sophisticated physical instruments. These values are typically not available during a real accident and were made available by the exercise organizers for comparison only. In some cases, the uncertainty is not given in the literature and, accordingly, could not be added to Table 1. For the calculations performed in the present paper, the uncertainty of *θ* is assumed to be equal to 0.02.

## Discussion

In the present paper two methods are proposed to assess biodosimetric doses, which offer reasonable alternatives to the widely used iterative and Bayesian methods.

The first analytical method is a straightforward mathematical development of the iterative method. It substantially reduces the propagation of uncertainties effect and is generally faster. Moreover, it reduces the probability of errors because it only requires to solve a set of equations instead to perform a series of calculations. The second Q-B method is substantially different because of the implementation of prior functions. This may provide more realistic results, because the knowledge about *θ* (or *ρ*) is usually not very precise. Therefore, the Gaussian prior is usually used, but other priors can be also appropriate. Choice of the prior will however influence the uncertainty which can be non-symmetrical. This choice is sometimes necessary, due to the limited knowledge about *θ* (or *ρ*) where some additional information might increase the reliability of the deduced parameter value, in some range of values.

In the Q-B method, the influence of other prior distributions such as gamma, cauchy, beta and geometric distributions was also evaluated here. It turned out that for the same *θ* value (the maximum of the distribution) all those PDFs give comparable (practically the same) results. The only difference is the uncertainty that is caused by the shape of distribution—generally the uncertainty is larger when the PDF is wider.

**Fig. 3:**
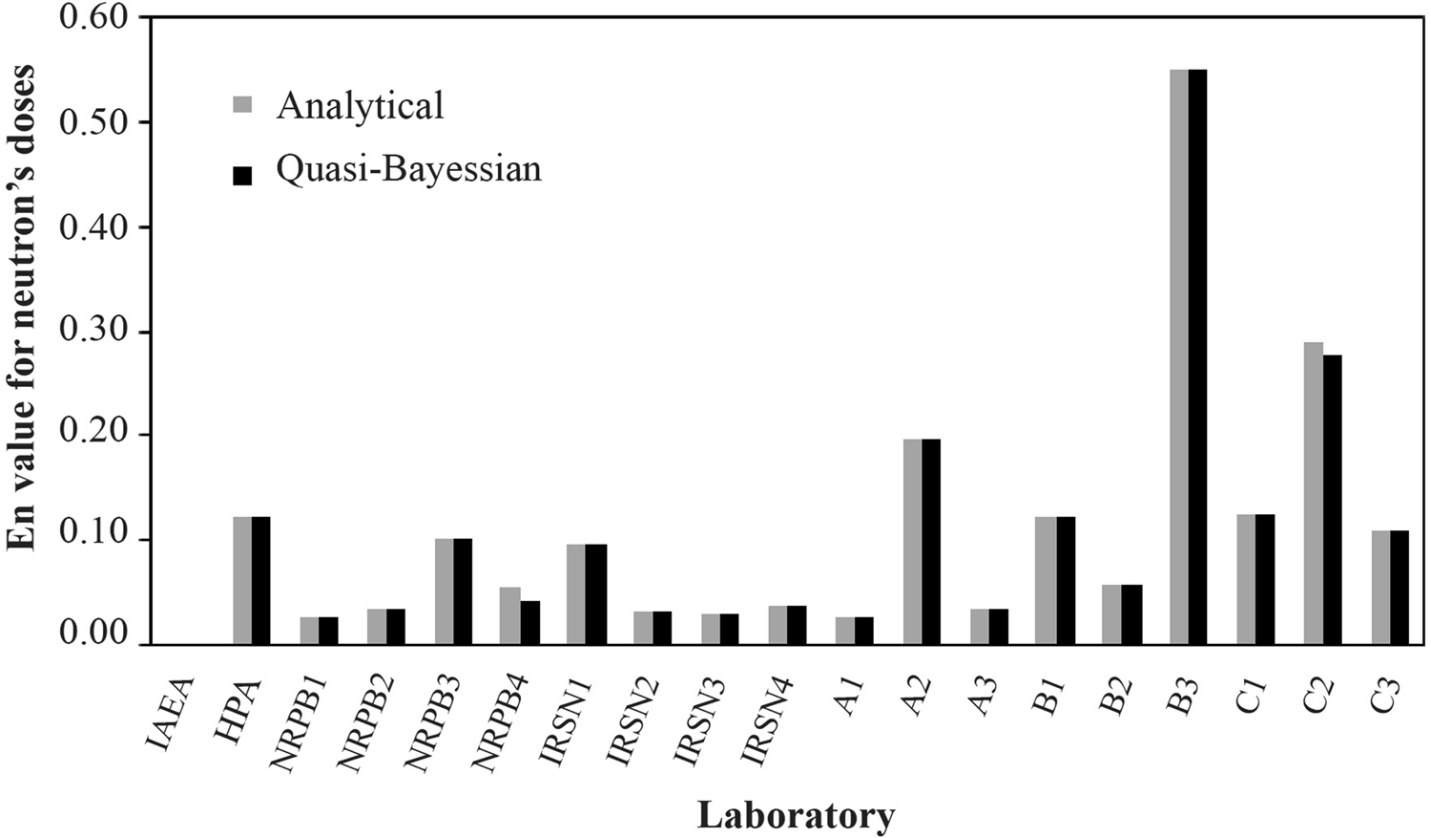
*E*_*n*_ values for neutron radiation

**Fig. 4:**
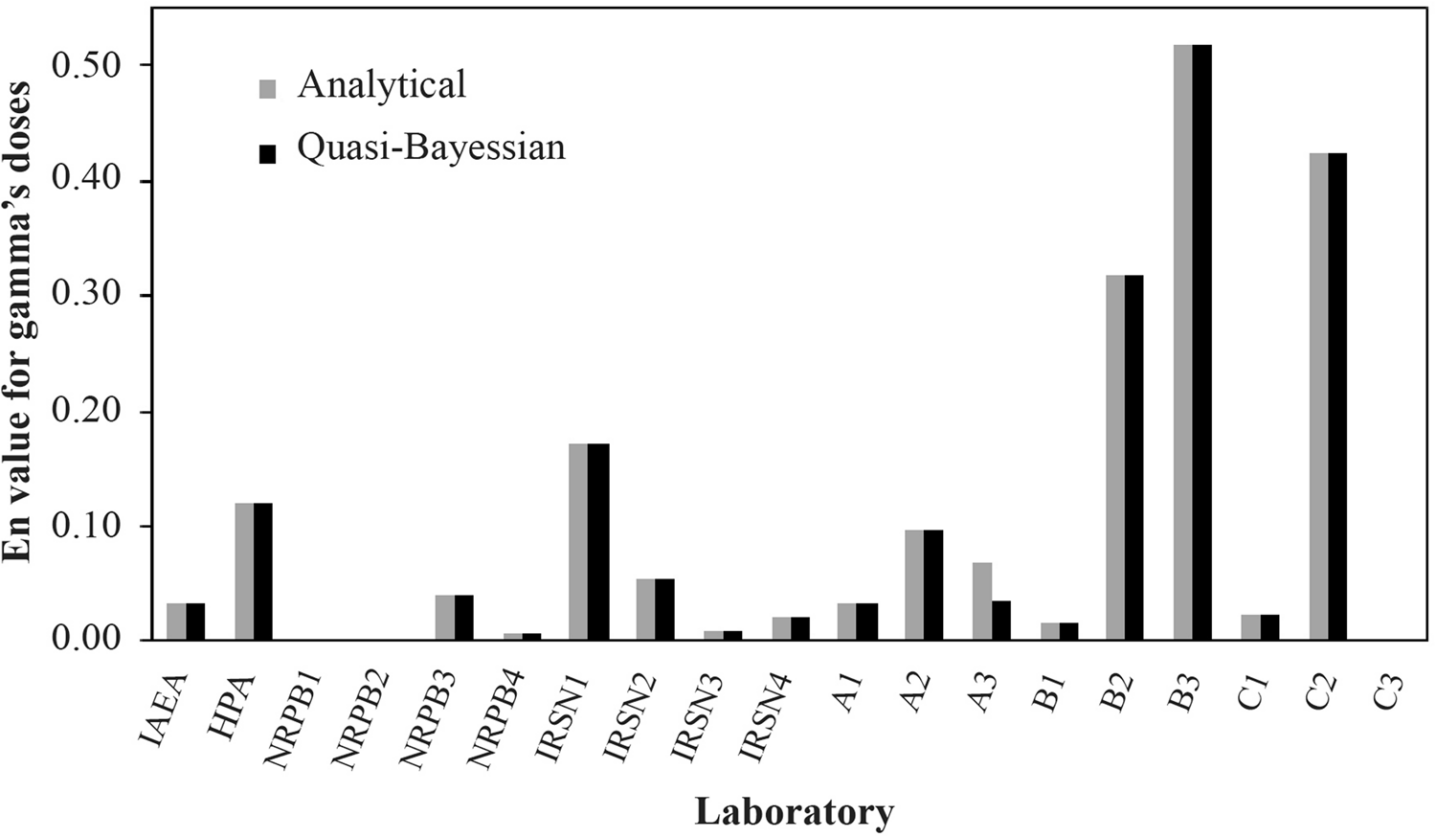
*E*_*n*_ values for gamma radiation

Table 1 shows doses obtained using both of the proposed methods, the analytical and the Q-B method with a Gaussian distribution, together with the original values from the literature. Additionally, the accuracy factor, *E*_*n*_, given by Eq. 17 provides a measure for the relative goodness of all assessments (Figs. 3, 4). For all results *E*_*n*_ is much below 1; this indicates only small deviations from the reference values. Thus, both methods provide results which are fully comparable with those the classical method. The biggest difference between the biological doses, and the doses obtained from the proposed methods using the Gaussian distribution as the PDF is visible for both the neutron and gamma doses, for samples A, B and C, especially B3 and C2. For sample B3, the *E*_*n*_ value equals 0.55 for the neutron dose, and 0.52 for the gamma dose. For sample C2 in the analytical method, *E*_*n*_ is 0.29 for the neutron dose, while for the gamma dose, equals 0.42. For quasi-Bayesian method it is 0.28 and 0.42, respectively. Interestingly, the above values do not seem to be caused by PDF selection and are rather method-independent. This is so especially for cases where the original dose uncertainties are unavailable and thus, an uncertainty of 10% *D*_*ref*_ had to be assumed in the calculations. Because Table 1 demonstrates that for samples A, B, and C there is also a quite significant difference between the biological and reference doses, it is concluded that the analytical and Q-B methods work also well for these cases, and large *E*_*n*_ values must have some other reasons.

It is generally not possible to identify one method as the best one, since the problem depends on the exact situation. While the analytical method provides almost identical point estimates as the Q-B method, the uncertainties obtained with the analytical method are slightly lower than those obtained with the Q-B method, which is probably due to the completely different methodology of both approaches, mostly the representation of *θ* as the PDF in the Q-B method. This might suggest that the uncertainties calculated by the Q-B method are more realistic than those calculated by the analytical method.

## Conclusion

In the case of mixed radiation exposure, the iterative method is widely used for biodosimetry. In the present study, two alternative statistical methods of estimating neutron and gamma absorbed doses in a mixed radiation field from measured chromosomal aberration frequencies were investigated. Both the analytical and the Q-B method proposed here have their own advantages and disadvantages. The first-proposed method is based on the classical iterative approach and is nothing more than its analytical description; for this reason the results obtained are the same, as shown in Table 1. Thus, the iterative method can be replaced by the analytical one which is more convenient in use because it does not need to perform a series of iterations; instead it only requires to insert the appropriate data into the given formulas. This classical method is a very simple and fast calculation method, but requires knowledge of the neutron/gamma dose ratio. In the Q-B method, this parameter is described by the probability distribution (prior function), similarly to Bayesian methods. Therefore, it can be used when the exact dose ratio is unknown without requiring complicated Bayesian statistics.

The statistical methods proposed in the present work were programmed as computational algorithms which can easily be used in cytogenetic analyses. Additionally, the methods are presented here in easy-to-use forms, which can be coded even as Excel spreadsheet formulas. The required computer codes are provided as an electronic supplement material.
